# Mindfulness-Based Stress Reduction in Pre-symptomatic Genetic Frontotemporal Dementia: A Pilot Study

**DOI:** 10.3389/fpsyt.2022.864391

**Published:** 2022-04-27

**Authors:** Jackie M. Poos, Esther van den Berg, Janne M. Papma, Fleur C. van der Tholen, Harro Seelaar, Laura Donker Kaat, J Anneke Kievit, Aad Tibben, John C. van Swieten, Lize C. Jiskoot

**Affiliations:** ^1^Department of Neurology and Alzheimer Center Erasmus MC, Erasmus MC University Medical Center, Rotterdam, Netherlands; ^2^Department of Clinical Genetics, Erasmus MC University Medical Center, Rotterdam, Netherlands; ^3^Department of Clinical Genetics, Leiden University Medical Center, Leiden, Netherlands; ^4^Dementia Research Centre, University College London, London, United Kingdom

**Keywords:** frontotemporal dementia (FTD), presymptomatic, mindfulness, depression, anxiety

## Abstract

Pre-symptomatic frontotemporal dementia (FTD) mutation carriers and first-degree family members that are 50% at-risk for FTD may experience symptoms of anxiety and depression as a result of the ambiguity of when or if symptoms of the disease will manifest. We conducted a pilot study to investigate the use of an online mindfulness-based stress reduction (MBSR) course to reduce symptoms of anxiety and depression in presymptomatic frontotemporal dementia (FTD) mutation carriers and individuals 50% at-risk. Seven known mutation carriers and six individuals 50% at-risk completed a standardized 8-week MBSR course, and filled out pre- and post and two-month follow-up questionnaires. The primary outcome measure was the Hospital Anxiety and Depression Scale (HADS). Measures of psychological distress (SCL-90-R), coping style (UCL), quality of life (SF-36) and mindfulness skills (FFMQ) were administered as secondary outcome. Group effects were analyzed with repeated measures ANOVA or Friedman's test, and the individual reliability change index (RCI) was calculated per participant for each outcome measure. Semi-quantitative data included an evaluation and process measure post-intervention. Significant decline was found on the HADS-A post-intervention and after 2 months (*p* = 0.01), with 54% and 62% of participants demonstrating a clinically significant RCI, respectively. On the HADS-D, significant decline was found 2 months post-intervention (*p* = 0.04), which was driven by 23% of participants whom had a clinically significant RCI. Additional changes were found between baseline and post-intervention on the seeking distraction and reassuring thoughts subscales of the UCL, the depression and interpersonal sensitivity subscales of the SCL, the observe subscale of the FFMQ, and on physical role limitations of the SF-36 (all *p* < 0.05). The process evaluation form indicated that the course was found beneficial by participants, and that they applied it in a wide range of everyday situations. This exploratory pilot study indicates the feasibility of MBSR in reducing anxiety and depression in presymptomatic FTD mutation carriers and 50% at-risk individuals. A randomized controlled trial is necessary to replicate these results.

## Introduction

Frontotemporal dementia (FTD) is an early-onset neurodegenerative disorder, associated with behavioral, cognitive, and/or motor impairment (1–3). It is a debilitating, fatal illness that has an autosomal dominant inheritance pattern in up to 30% of cases with high penetrance (4). Each child of an affected person has a 50% chance of inheriting the genetic mutation and thus developing the disease in the future. Age of symptom onset and prognosis varies within families, and there are currently no disease-modifying therapies available (5). Some individuals choose to undergo predictive testing to determine if they are carriers of a familial FTD mutation. However, knowledge of being a carrier represents unavoidable dementia onset and a dramatically shortened lifespan (5). Knowing one will develop a life-limiting condition, with possibly first-hand experience of the effects of the disease from one or multiple family members, may influence plans and attitudes toward the future, such as life planning, financial care and insurances (6, 7). It is therefore unsurprising that the ambiguity of being at risk for a neurodegenerative disorder with no cure may lead to a variety of adverse psychological reactions, such as anxiety and depression (8, 9).

Previous studies in other familial neurodegenerative disorders, such as familial Alzheimer's disease, Huntington's disease (HD) and Machado-Joseph disease, have indeed shown elevated depressive symptoms in presymptomatic mutation carriers aware of their genetic status, and individuals that are 50% at-risk of the disease (10–12). Very few studies have investigated psychological distress in genetic FTD mutation carriers, and were from a biological perspective, presuming that biological factors are the main determinants of neuropsychiatric symptoms in the early stage of FTD (13, 14). Yet, similar to what has been reported in premanifest HD individuals, reports from presymptomatic FTD mutation carriers often include emotional and social concerns such as anxiety about when symptoms will manifest, the impact of the disease on self and family, difficulties with acceptance of the disease, lack of support, perceived negative attitudes of others, and limited public awareness (15, 16). In the absence of disease-modifying treatment, psychological interventions are necessary that can reduce psychological distress experienced by individuals at-risk of developing FTD.

To our knowledge, no psychological interventions have been investigated in presymptomatic FTD mutation carriers that experience psychological distress, nor are there tailored therapeutic programs offered to these individuals in the Dutch healthcare system. Although multiple psychotherapeutic interventions are available for treating anxiety and depression, such as cognitive behavioral therapy (CBT), these approaches are based on the idea that distortions in thinking are the cause of psychological problems, and that efforts to change these thinking patterns can relief symptoms of anxiety/depression (17). Yet, the (often mild) psychological distress experienced by presymptomatic mutation carriers and 50% at-risk individuals is likely caused by the realistic scenario that one will or has a higher chance of developing a debilitating and fatal illness, rather than (unrealistic) distortions in thinking. A mindfulness-based approach could prove more beneficial to these individuals due to its underlying principles and practices. Mindfulness involves paying attention purposefully, in the present moment and without judgment (18). The focus is on cultivating conscious awareness on a moment-to-moment basis with an open and non-judgmental attitude by performing meditation-based exercises (18). Accepting things as they are, without trying to change them is emphasized (18). Hence, there has been an increasing interest in the application of mindfulness-based interventions, such as mindfulness-based stress reduction (MBSR) and mindfulness-based cognitive therapy (MBCT), in populations with chronic diseases (19, 20). Several studies have shown that such interventions are effective in cultivating acceptance of a long-term condition, and importantly that the social interaction with others that are in the same situation, in the form of group therapy rather than individual sessions, enhances the benefits of the intervention (21, 22). In other neurological diseases, e.g., Parkinson's disease and multiple sclerosis, mindfulness-based interventions have proven effective in lowering symptoms of anxiety and depression, and/or improving quality of life (23–25). Eccles et al. (6) have recently reported that MBCT was considered beneficial by premanifest HD individuals, but live sessions were not considered feasible due to recruitment issues as a result of the rarity of the disease. The authors suggested that online course delivery might be more feasible (6, 15). No feasibility or pilot study has been published on psychological approaches in known mutation carriers or 50% at-risk individuals of a mutation causative of FTD.

The present pilot study aimed to explore the feasibility and efficacy of a MBSR course in lowering psychological distress in known mutation carriers or individuals with a 50% risk of developing FTD. The primary aim was to investigate the effect of MBSR on reducing symptoms of anxiety and depression, and secondary aims were to investigate whether MBSR can reduce symptoms of psychopathology and stress, whether it can lead to a more beneficial coping style and whether it can improve health-related quality of life.

## Materials and Methods

### Participants

Fourteen participants were recruited *via* the FTD risk cohort study (FTD-RisC), in which cognitively healthy first-degree family members of patients with genetic FTD are longitudinally tracked (26). Participants had to be aged 18 or over. They had undergone predictive testing and were known mutation carriers of a *C9orf72, GRN, MAPT* or *TARDP* mutation, or were 50% at-risk (i.e., did not undergo predictive testing). They had to report experience of (mild) emotional burden, reflected in a Hospital Anxiety Depression Scale (HADS) score of ≥1 (27). Participants had to be asymptomatic according to established diagnostic criteria for bvFTD (3), PPA (2), and ALS (1), and have a CDR® plus NACC FTLD global score ≤ 0.5 (28). Clinical status was assessed as described previously (29). The Mini-Mental State Examination (MMSE) measured global cognitive functioning (30). Participant characteristics are given in Table 1. All participants' ethnicity was reported as Caucasian. Exclusion criteria included other neurological and/or psychiatric diagnoses, and participants had to attend at least six sessions of the MBSR training. Thirteen participants completed the MBSR training and one participant withdrew after session five. The latter missed three sessions early in the course due to personal circumstances and found it hard to immerse with the group and exercises after that. The study was approved by the Medical and Ethical Review Committee of the Erasmus MC University Medical Center (MEC-2019-0226).

**Table 1:** Participants characteristics.

*n*	13
Mean age (SD) [Range]	52.3 (11.7) [29–67]
Sex, ratio f:m	8:5
Mean education level^a^ (SD)	5.8 (0.8)
Known mutation carrier, yes:no	7:6
MMSE [Range]	29.1 (1.3) [26–30]
CDR^®^ plus NACC FTLD global score	
0, *n*	9
0.5, *n*	4
Affected gene in family, *n*	
*C9orf72*	5
*GRN*	5
*MAPT*	2
*TARDBP*	1

*Data are presented as mean (standard deviation) unless otherwise specified*.

*SD, standard deviation; f, female; m, male; MMSE, Mini-Mental State Examination; CDR^®^ plus NACC FTLD, Clinical Dementia Rating scale plus National Alzheimer's Coordinating Center Frontotemporal Lobar Degeneration; C9orf72, chromosome 9 open reading frame 72; GRN, progranulin; MAPT, microtubule-associated protein tau; TARDBP, TAR DNA binding protein*.

a *Level of education was recorded using seven categories in accordance with the Dutch educational system (1 = <6 years of primary education to 7 = academic schooling)*.

### Recruitment

All participants of the FTD-RisC study that fulfilled inclusion and exclusion criteria (*n* = 130) were invited for the training *via* an e-mail invitation that informed them about the MBSR training. Potential participants could then contact the research team to hear more about the study, after which the mindfulness teachers (LCJ and JMP) contacted the potential participant by telephone to discuss what participation involved. Twenty participants reached out to the research team and indicated to be interested in the study, of which five participants dropped out due to logistic reasons (i.e., dates and times did not suit them, training location too far away), and one participant was excluded due to a diagnosis of major depressive disorder according to the DSM-V (31). We ran two separate groups with a maximum of eight individuals so that there would be time during the sessions for individuals to connect and share experiences and stories.

### Procedure

All participants gave written informed consent. Primary and secondary outcome measures, as described in Section Outcome measures, were administered pre- and post-intervention, as well as 2 months after ending the course (Supplementary Table S1). Directly post-intervention participants were asked to fill out an evaluation form that was designed specifically for the purpose of this study (Section Intervention). Participants completed the questionnaires from home *via* the online survey tool LimeSurvey (32). The first three sessions of the first group were in person, but due to lockdown restrictions as the result of the COVID-19 pandemic, all other sessions of the first group were held online *via* Microsoft Teams. Due to positive reactions from the first group on the online course and in order to allow recruitment of people from a larger geographical area, all sessions of the second group were held online. Because of possible emotional reactions that may arise during the course, participants were invited to contact the MBSR teachers for any needs, questions, or practice support at any time outside the class setting.

### Intervention

The MBSR training followed the standard 8-week MBSR program which included meditation, mindful movement and yoga exercises, and information on the physiological and psychological basis of stress (33). An overview of the MBSR program per session is given in Supplementary Table S2. Each session lasted 120 min with a 15 min break. An all-day silent retreat as part of the standard program was not possible due to COVID-19 restrictions, and therefore we merged this aspect of the training with session 7 (Supplementary Table S2). Participants were asked to complete at least 45 min of daily home practice using provided audio fragments and worksheets that included stories, poetry and metaphors. This also included a personal log where participants were asked to fill out whether they performed the exercises. The course was taught by a certified MBSR trainer (LCJ) and an experienced MBSR practitioner (JMP), whom are both neuropsychologists with extensive experience in (presymptomatic) FTD.

### Outcome Measures

The primary outcome measure was the total, depression subscore (HADS-D) and anxiety subscore (HADS-A) of the HADS, developed to measure psychological distress in somatic patient populations (27, 34, 35).

In addition, five secondary outcome measures were included: the Symptom Checklist 90 Revised (SCL-90-R) for measuring psychological problems and symptoms of psychopathology (36), the Utrecht Coping List (UCL) for measuring coping styles (37), the 36-item Short Form Health Survey (SF-36) for measuring health-related quality of life (38), the Perceived Stress Scale (PSS) for measuring the perception of stress (39), and the 39-item Five Facet Mindfulness Questionnaire (FFMQ) for measuring mindfulness skills (40).

Further measurements included a visual analog scale (VAS) to measure the level of distress before and after each session, ranging from 0 (no distress) to 10 (very distressed) (41). To evaluate participants' experiences of the training they were asked to fill out an evaluation form that consisted of the Applied Mindfulness Process Scale (AMPS) (42), a process measure for evaluating mindfulness-based interventions, and 10 additional questions that focused on (1) the satisfaction with the MBSR training; (2) whether it helped them cope better with the higher risk of FTD; and (3) in case of group 1, their opinions on the (change to) online course. In addition, an open-ended box was added where participants were asked to share their experiences with the course.

### Statistical Analysis

Statistical analyses were performed in R version 4.04. The significance level was set at *p* < 0.05 (2-tailed) across all comparisons. There was no missing data.

#### Group Level

Parametric repeated measures analysis of variance (i.e., *F*-test statistic) or, in case of violated assumptions, non-parametric Friedman's tests (i.e., χ^2^ test statistic) were performed with the primary and secondary outcome measures (Section Outcome measures) as dependent variables and a within factor consisting of three time-points (i.e., baseline, post-intervention and 2 month follow-up). We performed pairwise comparisons between time points with parametric paired sample *t*-tests or non-parametric Wilcoxon signed-rank tests.

#### Individual Level

To determine clinically significant change in individual participants, the individual reliable change index (RCI) was calculated according to the Jacobson-Truax (1991) formulae using the JTRCI package in R (43). More specific, the RCI was calculated by dividing the absolute difference between the pre- and post-measurement by the standard deviation of the standard error of measurement for a difference score (Sdiff). The test-retest reliability coefficients were extracted from validation studies (35–37, 44–46). This study investigated mild symptoms of anxiety and depression in individuals that are a known mutation carrier or 50% at-risk of carrying a mutation causative of FTD; therefore we defined reliable change as ± 1 Sdiff.

## Results

### Quantitative Data

#### Primary Outcome Measure

**Figure 1:**
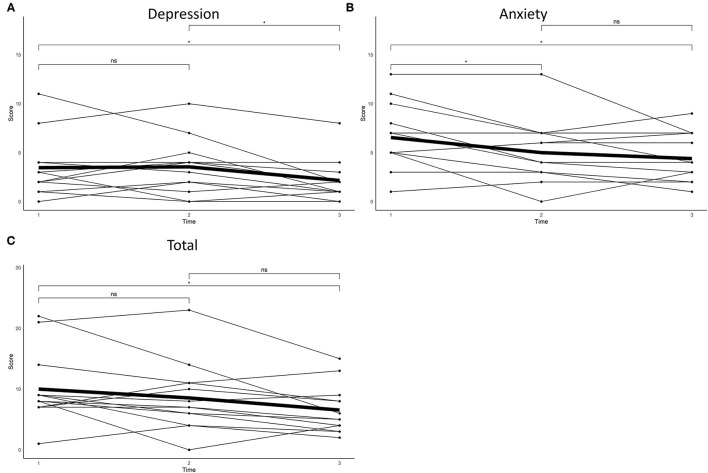
Self-reported **(A)** anxiety, **(B)** depression and **(C)** total scores on the Hospital Anxiety and Depression Scale at baseline, post-intervention and after 2 months.

The means and standard deviations of the HADS per time point are reported in Table 2. There was a significant difference between time points on the depression [*F*_(2,24)_ = 5.54, *p* = 0.01, ηp^2^ = 0.09; Figure 1A], anxiety [$\chi_{\left(2\right)}^{2}$ = 6.45, *p* = 0.04, W = 0.25; Figure 1B] and total [*F*_(2,24)_ = 4.87, *p* = 0.02, ηp^2^^=^ 0.07; Figure 1C] score of the HADS. Significant differences between time-points are illustrated in Figure 1.

**Table 2:** Mean, standard deviations per time point on the Hospital Anxiety and Depression Scale.

	**Baseline**			**Post-intervention**			**Two months follow-up**		
	**Total**	**Known carriers**	**50% at-risk**	**Total**	**Known carriers**	**50% at-risk**	**Total**	**Known carriers**	**50% at-risk**
Anxiety	6.54 (3.31)	6.71 (4.07)	6.33 (2.50)	5.00 (3.24)	5.00 (4.24)	5.00 (1.90)	4.38 (2.53)	4.14 (2.12)	4.67 (3.14)
Depression	3.46 (3.02)	4.14 (3.98)	2.67 (1.21)	3.54 (2.79)	4.14 (3.44)	2.83 (1.83)	2.15 (2.12)	2.00 (2.71)	2.33 (1.37)
Total	10.00 (5.80)	10.90 (7.73)	9.00 (2.61)	8.54 (5.67)	9.14 (7.63)	7.83 (2.48)	6.54 (3.95)	6.14 (4.38)	7.00 (3.74)

*All data are presented as mean (standard deviation)*.

**Figure 2:**
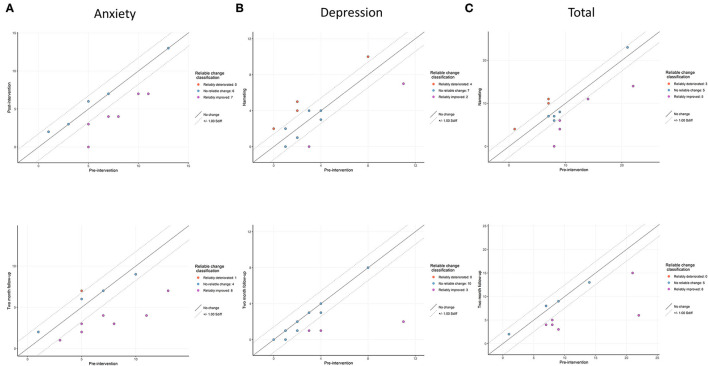
Reliable change indices on **(A)** anxiety, **(B)** depression and **(C)** total scores of the Hospital Anxiety and Depression Scale. The upper window represents the change between baseline and post-intervention and the lower window the change between baseline and after 2 months.

On the HADS-A, seven participants reported a clinically meaningful decline directly post-intervention, of whom 57% were known mutation carriers. Two months post-intervention eight participants reported a clinically meaningful decline, of whom 63% were known mutation carriers, whereas one participant, a known mutation carrier, reported an increase (Figure 2A). On the HADS-D, two participants reported a clinically meaningful decline directly post-intervention, whom were both known mutation carriers. Four participants reported an increase directly post-intervention, of whom 75% were known mutation carriers. After 2 months, three participants reported a decline, whom were all known mutation carriers (Figure 2B). Overall, five participants reported a clinically meaningful decline on the total score of the HADS, of whom 60% were known mutation carriers. Three participants reported an increase directly post-intervention, of whom 66% were known mutation carriers. After 2 months, eight participants reported a clinically meaningful decline, of whom 63% were known mutation carriers (Figure 2C).

#### Secondary Outcome Measures

The data on the secondary outcome measures are reported in Supplementary Table S3. Significant increases between time-points were found on the seeking distraction [*F*_(2,24)_ = 3.48, *p* = 0.05, ηp^2^ = 0.06] and reassuring thoughts [*F*_(2,24)_ = 8.3, *p* < 0.01, ηp^2^ = 0.12] subscales of the UCL, the observe [*F*_(1,15)_ = 7.51, *p* = 0.01, ηp^2^ = 0.15] and non-reactivity to inner experience [*F*_(1,15)_ = 4.52, *p* = 0.04, ηp^2^ = 0.14] subscales of the FFMQ, and physical [$\chi_{\left(13\right)}^{2}$ = 6.09, *p* = 0.05, W = 0.23] and emotional role functioning [$\chi_{\left(13\right)}^{2}$ = 6.74, *p* = 0.03, W = 0.26] of the SF-36. A significant decline was found on the depression [$\chi_{\left(13\right)}^{2}$ = 8.92, *p* = 0.01, W = 0.34], interpersonal sensitivity [$\chi_{\left(13\right)}^{2}$ = 13.7, *p* < 0.01, W = 0.53] and total psychoneuroticism [$\chi_{\left(13\right)}^{2}$ = 7.41, *p* = 0.03, W = 0.29] scores of the SCL-90-R and the general health perception [*F*_(2,24)_ = 3.47, *p* = 0.05, ηp^2^ = 0.12] subscale of the SF-36. *Post-hoc* pairwise comparisons between time-points revealed significant differences between baseline and post-intervention, except for non-reactivity to inner experience of the FFMQ, and emotional role functioning and general health perception of the SF-36, which were different between baseline and 2 months post-intervention. Calculation of the individual RCIs demonstrated that 2 months post-intervention more than 40% of participants increased on the seeking distraction (43% known mutation carrier) and reassuring thoughts (50% known mutation carrier) subscales of the UCL, the observe subscale of the FFMQ (67% known mutation carrier) and on the PSS (67% known mutation carrier) (Supplementary Table S3). Three participants reported an increase on the depression subscale of the SCL-90-R, of whom two were known mutation carriers. More than half of the participants reported a decline on general health perception of the SF-36 2 months post-intervention (71% known mutation carrier) (Supplementary Table S3).

### Semi-quantitative Data

#### Visual Analog Scale for Distress Level

Overall, participants reported a decline in distress level after each training session on the VAS with a mean delta of −1.94 and a standard deviation of 2.14. Only one participant experienced a strong increase in distress from 0.5 to 9 during session six. This was self-reported due to the mountain meditation at the end of that session, which reminded the participant of a recent personal stressful situation, but disappeared a few hours after the session.

#### Evaluation Form

The frequencies that participants answered “mostly true” and “often” or higher on each item of, respectively, the evaluation form and the AMPS are reported in Table 3. All participants (*n* = 13) indicated that they looked back on the course feeling satisfied, that they wanted to continue applying the exercises and skills learned in the course, and that they would recommend the course to others. All but one participant indicated that the course met their expectations and that they underwent the course at the right moment in their lives. Most participants (*n* = 11) felt that the course fitted in well with their daily life activities and liked that the course was offered online. All participants from group 1 indicated that they were not affected by switching from physical to online sessions. Fewer participants reported that the course was relevant in dealing with fears and uncertainty with respect to FTD (*n* = 9) and that the support and experiences from other participants helped them (*n* = 8).

**Table 3:** Results from the evaluation form and AMPS post-intervention.

**Evaluation form**	
**Statement**	**% ≥Mostly true**
I look back on the mindfulness course with a satisfied feeling	100%
The mindfulness course met my expectations	92.4%
The mindfulness course fitted in well with my daily activities and obligations	84.6%
The mindfulness course was at the right time in my life for me	92.4%
The mindfulness course was relevant to me in dealing with my fear and uncertainty about FTD	69.3%
The support and experiences on FTD from my fellow students helped me a lot	61.5%
I want to continue to apply the exercises and skills I learned in the mindfulness course	100%
I would recommend the mindfulness course to others	100%
I liked that the course was partly offered online due to the COVID pandemic	84.6%
I have not been affected by the switch from physical to online meetings due to the COVID pandemic	77%
**Applied Mindfulness Process Scale**	
I have used mindfulness practice to…	% ≥*often*
…view my thoughts from a distance	61.5%
…physically relax	84.6%
…realize that my thoughts do not have to be true	76.9%
…enjoy the little things more	84.6%
…calm myself down when I was feeling upset	84.6%
…not give into negative feelings right away	61.5%
…view a difficult situation from the positive side	84.6%
…reduce tension when I was feeling stressed	100%
…realize that I can grow stronger from negative situations	46.2%
…stop my unhelpful reactions to certain situations	69.2%
…notice and appreciate pleasant situations	92.4%
…put aside unpleasant thoughts or feelings	61.5%
…realize that my thoughts are not facts	84.6%
…notice the pleasant things in difficult situations	61.5%
…learn that there are other ways to look at certain situations	69.2%

*Answer options on the evaluation form were: not true, mostly not true, neutral, mostly true and true. Answer options on the AMPS were: never, rarely, sometimes, often and almost always. Percentages reflect the relative number of individuals that answered mostly true and often or higher*.

On the process measure scale, the two most reported daily-life situations where mindfulness skills were applied were to reduce tension when feeling stressed (*n* = 13) and to notice and appreciate pleasant situations (*n* = 12). This was followed in frequency by reports of having used mindfulness to physically relax, enjoy little things more, calm down when feeling upset, view difficult situations from the positive side and realize that thoughts are not facts (*n* = 11). Ten participants reported to have used mindfulness practice to realize that their thoughts are not necessarily true, and nine participants used it to stop unhelpful reactions and learn that there are other ways to look at difficult situations. More than half of the participants reported that they used mindfulness practice to view their thoughts from a distance, not give into negative feelings right away, and notice pleasant things in difficult situations (*n* = 8). Only six participants indicated that they used it to realize that they can grow stronger from negative situations.

In addition, four themes were identified from the open-ended box at the end of the evaluation form:

(1) increased awareness e.g.,

“*Before the course a walk outside was nice. Now I experience my surroundings more intensely. I smell the air, feel my legs, enjoy the colours that I see.”*

“*I have become more aware of everything around me. I am less often doing things on “autopilot”*.

(2) stress management e.g.,

“*The course has taught me tools to cope with stress. I have learned to put my situation into perspective. There are worse things that could happen. I am more observant of and accepting towards certain situations.”*

(3) contact with others in the same situation e.g.,

“*Meeting others with similar experiences, and hearing their stories has helped me a lot. It strengthens me to know that I am not alone.”*

(4) delivery mode e.g.,

“*The contact with fellow individuals at-risk for FTD was not completely successful for me due to the online aspect of the course, which was a disappointment.”*

“*It is a shame that we were not able to come together in person. However, I liked that I did not have to travel.”*

## Discussion

The primary aim of this pilot study was to explore the feasibility and efficacy of a MBSR course in lowering symptoms of anxiety and depression in individuals that are 50% at-risk or known mutation carriers of autosomal dominant FTD. Quantitative analyses demonstrated lower levels of anxiety on the HADS directly and 2 months post-intervention, and lower levels of depression 2 months post-intervention. Consistently, secondary analyses revealed a decline in depression and interpersonal sensitivity. Furthermore, participants reported to be more observant of their surroundings, to use the coping styles seeking distraction and reassuring thoughts more regularly, and to feel less restricted by role limitations due to physical health. An evaluation and process measure form indicated that participants were overall satisfied with the MBSR course, and that they applied mindfulness skills in a wide range of daily activities. Taken together, the results of this exploratory pilot study indicate that an online MBSR course could be a feasible intervention for reducing symptoms of anxiety and depression in 50% at-risk individuals or known mutation carriers of autosomal dominant FTD.

Anxiety scores on the HADS declined at the first post-intervention measurement, and remained significantly lower 2 months after ending the MBSR program. Individual RCI calculations demonstrated that more than 60% of participants reported a clinically meaningful decline in anxiety level. These findings are consistent with the conceptual focus of the intervention: mindfulness principles focus on letting thoughts come and go easily, without attempting to alter, diminish or expand them (47). Through MBSR participants learn to view their mental events (such as anxiety or stress) as transient, and not reality (47). Eccles et al. (6) investigated the use of a mindfulness program in preclinical HD individuals, and suggested that mindfulness can help anchor individuals in the present rather than allowing fear to drive them into the future (6). Qualitative results indicated that participants used mindfulness skills in everyday life, for example, to realize that thoughts are not necessarily true or factual, to not give in to negative feelings, to put aside unpleasant thoughts and feelings, to learn that there are other ways to look at certain situations and to put their situation (i.e., being at-risk for or a known mutation carrier of FTD) in a new perspective, which all potentially could have contributed to less anxious feelings and thoughts.

Depression scores on the HADS were not lower directly post-intervention, but they were lower after 2 months post-intervention. This significant group effect appeared to be driven by two to three participants that reported a clinically meaningful change directly post-intervention and after two months. Most participants remained unchanged and, surprisingly, four participants reported an increase post-intervention which was no longer present after 2 months. A possible explanation for this result is that, a floor effect was observed in ~50% of participants, as only two participants reported a HADS-D score higher than four at baseline. Due to the low variation in test scores, significant RCIs were observed in those participants that reported only small changes in test scores. Studies in other preclinical neurodegenerative populations have also been contradicting, with some reporting higher levels of depression in at-risk individuals, whereas other studies were unable to confirm this (8–12, 48–55). One hypothesis is that the psychological distress experienced by known mutation carriers or 50% at-risk individuals for FTD mostly stems from stress-related and anxious feelings about an uncertain future rather than mood-related problems. Another hypothesis is that the complexity of feelings and emotional distress experienced by individuals that are at-risk of a life-limiting condition cannot be expressed in a quantitative measure such as the HADS. The HADS measures levels of anxiety and depression in the past 4 weeks, whereas the psychological distress that presymptomatic mutation carriers, and even individuals at 50% risk, experience is caused by a transient situation (i.e., they remain a mutation carrier or 50% at-risk for a mutation). This raises the question whether mood-related problems that arise as a result of being at-risk can be captured with the HADS, or any quantitative measure. Interestingly, a significant decline from pre- to post-intervention and after 2 months was found on the depression subscale of the SCL-90-R, which appeared to be driven by three, partly different, participants that reported a clinically meaningful change. This suggests that the course was effective in lowering depressive symptoms for some individuals. Specifically known mutation carriers appeared to benefit from the intervention in lowering depressive symptoms as five out of six participants that reported a clinically meaningful decline on either the HADS-D or the SCL-90-R had undergone predictive testing and were found to carry a genetic mutation. This is possibly due to most of them having higher depression scores at baseline than individuals that are merely 50% at-risk. This heterogeneity in our sample might have influenced results on other outcome measures as well. Qualitative interviews with people at-risk for FTD might shed more light on the variety of adverse psychological reactions that they experience and can help identify a suitable outcome measure for a future randomized controlled trial (RCT).

A decrease in interpersonal sensitivity was observed on secondary outcome measures. This is in line with a previous study that showed that mindfulness traits are negatively correlated with interpersonal sensitivity (56). Furthermore, an increase in reassuring thoughts and seeking distraction as coping styles was observed. It has been suggested that the central themes within a MBSR course, such as acceptance of thoughts and feelings and non-judgmental awareness to them, can show new ways to respond and cope with internal and external problems as well as help decrease habitual problematic patterns of thinking, feeling and behavior (47). By shifting their thoughts' focus toward calming or tranquil thoughts, thereby reinforcing positivity, helps participants in realizing how unhelpful negative thoughts and feelings are. This was also reflected by the change in how limited they felt by physical role limitations on the SF-36, and by what was reported on the process measure scale (e.g., physically more relaxed, enjoy little things more, view difficult situations from the positive side, notice and appreciate pleasant situations). Surprisingly, only the observe subscale of the FFMQ significantly increased post-intervention, indicating that the other mindfulness facets did not change as a result of the course. Consistently, participants reported to have become more aware of their surroundings on the evaluation form. A possible explanation for why we did not find improvement on the other facets is that the group mean for non-judging to inner experience, acting with awareness and describing was at baseline already similar to that of experienced meditators, possibly causing a ceiling effect (57). In contrast, the group mean for observing and non-reacting to inner experience were comparable to a non-meditating sample at baseline, allowing improvement over time (57). Lastly, a change over time was observed on the emotional role restrictions and general health perception subscales of the SF-36. However, this effect was only visible 2 months post-intervention and it seems therefore more likely that these changes were caused by different health-related life events or the lower test retest reliability that has been reported previously on specifically these subscales (44).

All sessions led to a decrease in acute stress levels as measured by a visual analog scale. Furthermore, qualitative data indicated that the MBSR course was found beneficial by participants and that they wanted to continue applying what they had learned in their daily lives, which, according to what was reported on the process measure, included a wide range of activities. Most participants indicated that they liked the online aspect of the course, as it did not require them to travel far. Although some individuals indicated that it helped them to meet others with similar experiences, others indicated that they missed this specific aspect due to the online sessions and that they would have preferred to meet in person. Meeting online did allow us to recruit people from a larger geographical area.

To our knowledge this is the first pilot intervention study in individuals at-risk for and carriers of a gene mutation causative of FTD. There are a few limitations to this study that should be taken into account when interpreting the results. First, as this was an exploratory pilot study to determine the feasibility and efficacy of the intervention, no sample size calculations were performed and only a small sample of individuals were recruited. Furthermore, the high number of outcome measures may have increased the family-wise error rate in our data. However, we emphasize the exploratory nature of our study and therefore lack of correction for multiple comparisons. For these reasons, quantitative analysis of questionnaires should be interpreted cautiously. Secondly, no control group was included as we did not have access to a large enough cohort of mutation carriers or at-risk individuals that experience psychological distress. It is therefore not possible to infer the specific effect of MBSR on lowering symptoms of anxiety and depression. A multi-center RCT that compares to an active control group as well as a larger sample size (e.g., within the Genetic FTD Initiative (GENFI)) is necessary to replicate the results from this pilot. Thirdly, most individuals from our sample of at-risk individuals and presymptomatic mutation carriers scored in the ‘normal' range compared to a reference population on all outcome measures, likely resulting in floor and ceiling effects. A possible moderating factor could be estimated time to (potential) symptom onset, with older individuals experiencing different emotions and thoughts related to FTD than younger individuals. Inclusion criteria for a RCT should be carefully considered. Furthermore, future research should focus on developing and validating other outcome measures that cover the psychological distress experienced by individuals at-risk of a genetic form of dementia better. Lastly, due to the COVID-19 pandemic we had to switch from in-person to online meetings after session three of the first group. We cannot directly compare this online version of MBSR with the face-to-face version of the intervention, but it could be that the online structure of the MBSR course is less effective than a live one as it might have negatively impacted bonding and support between participants. A recent systematic review revealed medium positive effects of online MBSR/MBCT delivery on mental health outcomes compared with inactive controls, and little difference with active controls such as in-person delivery (58). However, most included studies in the review had low methodological quality (58) and thus further studies to compare different delivery options in this field are necessary.

To conclude, this exploratory pilot study indicates the feasibility of online MBSR in individuals 50% at-risk and known carriers of a mutation causative of FTD in reducing symptoms of anxiety and depression. A randomized controlled trial in this population is necessary to confirm these results.

## Data Availability Statement

The raw data supporting the conclusions of this article will be made available by the authors upon reasonable request.

## Ethics Statement

This study was reviewed and approved by the Medical and Ethical Review Committee of the Erasmus University Medical Center. All participants provided their written informed consent to participate in this study.

## Author Contributions

JPa, LJ, EB, JPo, AT, and JS: concept design. JPo and LJ: data collection. JPo and FT: statistical analysis. JPa and JPo: drafting of the manuscript, and revision of the manuscript. All authors data interpretation and revising the manuscript. All authors contributed to the article and approved the submitted version.

## Funding

This work was supported by the Dioraphte Foundation [grant numbers 09-02-00], the Association for Frontotemporal Dementias Research Grant 2009, The Netherlands Organization for Scientific Research (NWO) grant HCMI [grant number 056-13-018], ZonMw Memorabel (Deltaplan Dementie), [project numbers 733 050 103 and 733 050 813], JPND PreFrontAls Consortium project number 733051042, and Alzheimer Nederland and the Bluefield project.

## Conflict of Interest

The authors declare that the research was conducted in the absence of any commercial or financial relationships that could be construed as a potential conflict of interest.

## Publisher's Note

All claims expressed in this article are solely those of the authors and do not necessarily represent those of their affiliated organizations, or those of the publisher, the editors and the reviewers. Any product that may be evaluated in this article, or claim that may be made by its manufacturer, is not guaranteed or endorsed by the publisher.
