# Supplementary material for: Mindfulness-Based Stress Reduction in Pre-symptomatic Genetic Frontotemporal Dementia: A Pilot Study
Source: Front Psychiatry. 2022 Apr 27;13:864391. doi: 10.3389/fpsyt.2022.864391 (PMC9091907; doi:10.3389/fpsyt.2022.864391)
Supplement: Supplementary file 1 [file Table_1.DOCX]

Supplementary Material

# Supplementary Data

Table S1. Outcome measures and assessments during MBSR programme.

| **Measure** | **Target** | **Baseline** | **Post-intervention** | **2 months**  **follow-up** |
| --- | --- | --- | --- | --- |
| *Primary* | | | | |
| HADS | Psychological distress | x | x | x |
| *Secondary* | | | | |
| SF-36 | (Health-related) Quality of life | x | x | x |
| UCL | Coping | x | x | x |
| SCL-90-R | Psychological problems and symptoms of psychopathology | x | x | x |
| PSS | Stress | x | x | x |
| FFMQ | Mindfulness skills | x | x | x |
| *Other* | | | | |
| VAS Distress thermometer | Psychological distress | Before and after each training session | | |
| Calendar | Mindfulness adherence | Monthly during study period | | |
| Evaluation form | Evaluation of the training aspects process measure for evaluating mindfulness-based interventions | Only post-intervention | | |

Table S2. Overview of MBSR program per session.

| **Week** | **Theme of session** | **Exercises** | **Didactic teaching** | **Homework** |
| --- | --- | --- | --- | --- |
| 1 | Automatic pilot (living in the present, not the insecure future) | - Body scan | - Intention of participating - Introduction mindfulness - Raisin exercise | - Body scan - Eating one meal mindfully - Attention for routine activity |
| 2 | Dealing with obstacles | - Body scan - Sitting meditation | - Awareness of pleasant and unpleasant events - Imaginary exercise to demonstrate relationship between thoughts and feelings - The seven essential attitudes of mindfulness | - Body scan - Attention for breathing - Awareness of pleasant events - Attention for routine activity |
| 3 | Observing your limits, recognizing signs from your body | - Yoga while lying down - 3 minute breathing space | - Breathing as an anchor for attention | - Body scan or yoga - Sitting meditation - Awareness of unpleasant events - 3 minute breathing space |
| 4 | Opening up to distress | - Meditation on hearing - Yoga while standing - Sitting meditation | - Interrelatedness of feelings, thoughts, and bodily sensations - Psychoeducation about stress | - Sitting meditation or yoga - Awareness of stressfull events - 3 minute breathing space |
| 5 | Responding to stress | - Sitting meditation - Walking meditation - 3 minute breathing space | - Psychoeducation about stress - Reacting vs. responding - Introducing silent session | - Sitting meditation and walking meditation (interchangeably) - Awareness of reaction in difficult situation - 3 minute breathing space |
| 6 | Thoughts are no facts | - Mountain meditation | - Exercise focus on something difficult - Exercise automatic negative thoughts | - Sitting meditation or yoga - Awareness of automatic negative thoughts - 3 minute breathing space during stress |
| 7 | Here and now (silent session) | - Sitting meditation - Yoga while lying down - walking meditation |  | - Own programme - Daypart in silence |
| 8 | Taking care of yourself | - 3 minute breathing space - Body scan - Short sitting meditation | - Exercise energy givers vs. energy takers - Letter to your future self - Evaluation - How to keep mindfulness in your life | - Further sources of information |

Table S3. Mean, standard deviations and percentage of participants that had a reliable in- or decrease per time point on the secondary outcome measures.

|  | **Pre-intervention** | | **Post-intervention** | | | | **Two months follow-up** | | | |
| --- | --- | --- | --- | --- | --- | --- | --- | --- | --- | --- |
|  | M | SD | M | SD | + | - | M | SD | + | - |
| **Utrechtse Coping List** | | | | | | | | | | |
| Active coping | 11.80 | 3.10 | 12.20 | 3.47 | 15 | 8 | 13.40 | 3.15 | 31 | 8 |
| Seeking distraction | 8.54 | 2.70 | 10.10 | 3.52 | 39 | 8 | 10.20 | 2.58 | 54 | 8 |
| Avoidance | 9.23 | 2.52 | 9.62 | 1.94 | 0 | 0 | 10.10 | 2.81 | 31 | 8 |
| Seeking social support | 8.54 | 3.43 | 8.85 | 3.21 | 15 | 8 | 9.77 | 3.03 | 23 | 8 |
| Passive coping | 3.46 | 2.54 | 3.85 | 3.26 | 31 | 15 | 3.15 | 1.62 | 8 | 23 |
| Expressing emotions | 1.92 | 1.12 | 2.00 | 0.82 | 8 | 0 | 1.69 | 1.11 | 0 | 8 |
| Reassuring thoughts | 8.00 | 2.77 | 9.38 | 3.25 | 23 | 0 | 10.50 | 2.90 | 77 | 0 |
| **Symptom Checklist 90 Revised** | | | | | | | | | | |
| Anxiety | 4.62 | 5.42 | 3.62 | 3.28 | 23 | 0 | 2.62 | 2.40 | 15 | 0 |
| Agoraphobia | 2.46 | 4.41 | 1.46 | 2.50 | 23 | 8 | 1.15 | 2.27 | 23 | 0 |
| Depression | 10.4 | 10.1 | 6.62 | 6.36 | 23 | 0 | 4.69 | 3.75 | 23 | 0 |
| Somatization | 5.46 | 4.45 | 4.38 | 3.43 | 31 | 31 | 4.23 | 4.07 | 23 | 15 |
| Insufficiency | 6.69 | 5.45 | 4.77 | 2.77 | 23 | 0 | 4.23 | 2.46 | 31 | 0 |
| Sensitivity | 8.23 | 8.20 | 6.08 | 11.3 | 15 | 8 | 5.54 | 6.44 | 15 | 0 |
| Hostility | 1.38 | 1.71 | 1.00 | 1.78 | 23 | 8 | 0.62 | 1.19 | 31 | 0 |
| Sleeplessness | 2.85 | 2.88 | 2.23 | 2.80 | 8 | 8 | 2.08 | 2.56 | 15 | 0 |
| Total psychoneuroticism | 44.8 | 36.5 | 32.4 | 30.6 | 23 | 0 | 27.2 | 19.2 | 15 | 0 |
| **Five Facet Mindfulness Questionnaire** | | | | | | | | | | |
| Observe | 26.10 | 4.79 | 29.10 | 4.07 | 39 | 8 | 29.90 | 3.55 | 46 | 0 |
| Describe | 29.00 | 6.44 | 28.10 | 6.64 | 8 | 31 | 28.80 | 6.25 | 15 | 23 |
| Acting with awareness | 28.60 | 5.68 | 27.50 | 3.69 | 8 | 8 | 29.20 | 5.29 | 15 | 8 |
| Non-judging of inner experience | 29.50 | 5.44 | 30.20 | 5.86 | 8 | 8 | 30.80 | 4.42 | 15 | 8 |
| Non-reacting to inner experience | 22.50 | 5.36 | 24.50 | 2.22 | 23 | 0 | 25.90 | 2.87 | 39 | 0 |
| **36-item Short Form Health Survey** | | | | | | | | | | |
| Physical functioning | 29.20 | 0.90 | 28.30 | 2.50 | 8 | 39 | 28.90 | 1.26 | 8 | 23 |
| Social functioning | 9.08 | 1.19 | 9.54 | 0.88 | 8 | 0 | 9.69 | 0.48 | 23 | 0 |
| Physical role functioning | 7.00 | 1.47 | 7.23 | 1.48 | 15 | 8 | 8.00 | 0.00 | 31 | 0 |
| Emotional role functioning | 5.46 | 0.88 | 5.62 | 0.77 | 31 | 8 | 6.00 | 0.00 | 39 | 0 |
| Mental health | 22.40 | 4.74 | 23.90 | 3.73 | 39 | 8 | 24.30 | 2.93 | 23 | 0 |
| Vitality | 16.80 | 4.00 | 18.10 | 1.80 | 31 | 15 | 18.20 | 2.80 | 39 | 8 |
| Pain | 51.60 | 7.88 | 50.70 | 10.20 | 15 | 23 | 54.10 | 5.63 | 31 | 8 |
| General health perception | 18.60 | 2.84 | 18.20 | 3.29 | 15 | 23 | 16.40 | 1.76 | 15 | 54 |
| **Perceived Stress Scale** | | | | | | | | | | |
| Total | 28.70 | 3.40 | 28.50 | 2.60 | 23 | 15 | 27.40 | 1.85 | 46 | 31 |

+ indicates a reliable increase, - indicates a reliable decrease.
